# The spatio-temporal epidemic dynamics of COVID-19 outbreak in Africa

**DOI:** 10.1017/S0950268820001983

**Published:** 2020-09-02

**Authors:** Ezra Gayawan, Olushina O. Awe, Bamidele M. Oseni, Ikemefuna C. Uzochukwu, Adeshina Adekunle, Gbemisola Samuel, Damon P. Eisen, Oyelola A. Adegboye

**Affiliations:** 1 Department of Statistics, Federal University of Technology, Akure, Nigeria; 2 Population Study Center (NEPO), Universidade Estadual de Campinas, Campinas, Brazil; 3 Department of Mathematics, Anchor University, Lagos, Nigeria; 4 Institute of Mathematics and Statistics, Federal University of Bahia (UFBA), Salvador, Brazil; 5 Faculty of Pharmaceutical Sciences, Nnamdi Azikiwe University, Awka, Anambra State, Nigeria; 6 Australian Institute of Tropical Health and Medicine, James Cook University, Townsville, Australia; 7 Department of Demography and Social Statistics, Covenant University, Ota, Nigeria; 8 College of Medicine and Dentistry, James Cook University, Townsville, Australia

**Keywords:** Africa, Bayesian analysis, COVID-19, hurdle Poisson, spatial analysis

## Abstract

Corona virus disease 2019 (COVID-19), caused by the novel severe acute respiratory syndrome coronavirus 2 (SARS-CoV-2), was first detected in the city of Wuhan, China in December 2019. Although, the disease appeared in Africa later than other regions, it has now spread to virtually all countries on the continent. We provide early spatio-temporal dynamics of COVID-19 within the first 62 days of the disease's appearance on the African continent. We used a two-parameter hurdle Poisson model to simultaneously analyse the zero counts and the frequency of occurrence. We investigate the effects of important healthcare capacities including hospital beds and number of medical doctors in different countries. The results show that cases of the pandemic vary geographically across Africa with notably high incidence in neighbouring countries particularly in West and North Africa. The burden of the disease (per 100 000) mostly impacted Djibouti, Tunisia, Morocco and Algeria. Temporally, during the first 4 weeks, the burden was highest in Senegal, Egypt and Mauritania, but by mid-April it shifted to Somalia, Chad, Guinea, Tanzania, Gabon, Sudan and Zimbabwe. Currently, Namibia, Angola, South Sudan, Burundi and Uganda have the least burden. These findings could be useful in guiding epidemiological interventions and the allocation of scarce resources based on heterogeneity of the disease patterns.

## Introduction

On 11 March 2020, the World Health Organization (WHO) declared the novel coronavirus disease (COVID-19) outbreak a pandemic. The disease, caused by severe acute respiratory syndrome coronavirus 2 (SARS-CoV-2), was first reported in the city of Wuhan, China in late December 2019 and has quickly spread globally with 11 937 659 cases and a case fatality rate (CFR) of 4.57% as of 7 July 2020. [1] This pandemic has not only become a public health crisis leading to loss of life but has affected the global economy with severe disruption to international travel, tourism and trade [2]. As of 7 July 2020, all African countries combined have reported 494 380 confirmed cases and 11 652 deaths from the pandemic yielding a CFR of 2.36%. It is however, likely that case ascertainment in Africa is incomplete.

Human population movement generally plays an important role in the spread of infectious diseases and this particularly applies to COVID-19 as SARS-CoV-2 is highly transmissible. The reasons for the late appearance of COVID-19 in Africa compared with other parts of the world are unknown but it may be due to relatively limited international travel to the continent [3]. African countries predominantly reported their first COVID-19 cases to be imported from Europe [4]. The first confirmed case on the African continent on 14 February 2020 occurred in Egypt followed by Nigeria on 27 February 2020. The initial dynamics of the disease demonstrated a slow spread across the continent until the situation escalated abruptly in the last week of March.

Global experts have shown concern about the spread of COVID-19 in Africa, because of grossly underfunded and inadequate healthcare systems. Early detection and control of outbreaks is inefficient and unreliable due to poor disease surveillance, insufficient training of healthcare workers and inadequate data transmission [3, 5–8].

There have been a number of applications of statistical models for prediction of infection rates and spread during the COVID-19 pandemic [9, 10]. However, mapping of disease incidence to identify spatial clustering and patterns remains an important pathway to understanding disease epidemiology and is required for effective planning, prevention or containment action [11–13]. There are a few studies that attempt to map the pandemic in China [14] and in Iran [15]. However, the temporal dynamic of the COVID-19 pandemic has not been taken into account in order to assess space-time dynamics although a descriptive representation of the spatio-temporal pattern of the CFR was published for Brazil [16].

Our aim, therefore, is to analyse the spatio-temporal dynamics of COVID-19 within the first 62 days of the disease's arrival on the African continent. We propose a two-parameter hurdle Poisson model to simultaneously analyse the zero counts as well as average occurrence of the disease. The two parameters are extended, through appropriately chosen link functions, to the spatio-temporal covariates following the framework of distributional regression coined by Klein *et al*. [17]. Additionally, we investigate the effect of important healthcare capacities including hospital beds and the number of medical doctors on the risk of COVID-19 in the different African countries. The hurdle model is a modified count model in which two processes generating the zeros and the positives are not constrained to be the same [18]. The idea is that a binomial distribution model governs the binary outcome that stipulates whether the count variable returns a zero or a positive realisation and a Poisson distribution models the truncated-at-zero count data. With this, we are able to examine both the patterns of zeros and the average counts of the pandemic across space and time throughout Africa.

## Methods

### Data sources

We used publicly available daily number of confirmed COVID-19 cases reported by the World Health Organization (https://covid19.who.int) from 14 February to 15 April 2020. Due to the requirement of the spatial effect model considered in this study, we only included 47 African countries that have confirmed COVID-19 cases and share at least one international boundary. Additionally, we obtained data on healthcare capacities; number of hospital beds and physicians for each of the countries from the World Development Indicators of the World Bank (https://data.worldbank.org). Physicians include generalist and specialist medical practitioners while hospital beds include inpatient beds available in public, private, general, and specialised hospitals and rehabilitation centres. The most recent data for number of physicians per 1000 was from 2018 while that for hospital beds was from 2015.

### Statistical analysis

Preliminary exploratory spatial analysis was used to investigate the spatial and temporal distribution of incidence of COVID-19 cases and healthcare capacities (number of hospital beds and number of physicians) across Africa. We used Pearson's correlation to assess the relationships between the number of confirmed COVID-19 cases and each of the two healthcare capabilities of each country.

For the spatio-temporal analysis, we considered a two-component hurdle Poisson model which consists of a point mass at zero followed by a truncated Poisson distribution for the non-zero count observation. For an independently and identically distributed random variable, the hurdle Poisson distribution is expressed as
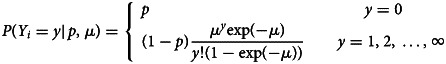
where *Y*_*i*_ is the response variable of interest that counts the number of reported cases of COVID-19 in a particular country, *p* is the none occurrence probability (the probability of not reporting a COVID-19 case in a given day) and *μ* measures the frequency of occurrence (the expected value of the Poisson distribution). Thus, as *μ* increases, the average count of COVID-19 increases. If *p* is 0, this implies that each country reported an infection during the period under consideration, but if *p* is 1 then there would be no infections caused by the pathogen on the continent during the period under consideration. Usually, *p* is considered to be strictly between 0 and 1, such that everyone in the population of the African continent has a non-zero probability of being infected with the virus even if they do not get infected during the period considered. Under the hurdle distribution, the expected value of *Y* is given by *E*(*Y*) = *pμ*/(1 − exp( − *μ*)).

Based on the framework of distributional regression that allows multiple parameters of a response distribution, rather than just the mean as is common in most classical applications, we extend the two parameters space 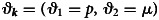 of the hurdle Poisson model to the spatial and spatio-temporal covariates of the COVID-19 cases in Africa. Suitable (one-to-one) link functions that ensure appropriate restrictions on the parameter space were considered.

The general form of the geo-additive hurdle Poisson model considered is given by
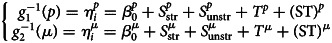
where *g*_1_ and *g*_2_ are link functions chosen as logit and log links for the parameters *p* and *μ*, respectively. Omitting superscript, *β*_0_ is the model constant term, *S*_str_ is the structured spatial random effect, *S*_unstr_ is the unstructured random effect, *T* is the temporal term and ST accounts for the spatio-temporal random effect. The structured component assumes a spatial correlation among the countries such that neighbouring countries are assumed to have more influence on one another than those far apart while the unstructured component assumes the countries are independent of one another. The temporal term was modelled based on a Bayesian P-spline. This allows for the estimation of the temporal term as a linear combination of basis spline (B-splines) based on a second-order random walk prior with inverse gamma for the hyperparameters [19]. We considered cubic B-splines with 20 equidistance knots, which allows enough flexibility to capture even the most severe non-linearity.

For the all structured spatial and spatio-temporal effects, countries are considered as discrete sets of spatial locations and we used a Markov random field prior that considers a binary structure for the neighbourhood structure of the countries such that proximate locations that share boundaries are assigned a weight of 1 and 0 if they do not. To ensure smoothness, we consider a Gaussian Markov random field prior that induces a penalty in which differences between spatially adjacent regions are penalised. An exchangeable independent and identically distributed normal prior was considered for the unstructured random effects. We provide more information on the estimation procedure of the model in Appendix 1 but details of geo-additive models including the different types of variables that can be included beyond those considered in this study and the specifics of prior distributions are contained in Fahrmeir *et al*. [20] and Fahrmeir and Kneib [21].

The Bayesian inference is based on the distributional regression framework of Klein *et al*. [17], who developed a Markov chain Monte Carlo simulation technique in which suitable proposal densities are constructed based on iterative weighted least-squares approximation to the full conditional. All smoothing variance parameters and hyperparameters were assigned inverse gamma hyperpriors. We performed sensitivity analyses but the results, based on the different hyperpriors, turned out to be indistinguishable.

To implement the spatio-temporal component, the complete spatio-temporal data were grouped into six periods: the first period represents the first month (due to paucity of data), and the remaining data are aggregated into one week periods. The intention was to examine how the countries fared in terms of the occurrence of the pandemic over a weekly period. We implement four models, by sub-setting temporal and spatial covariates on the mean parameter while keeping the temporal, structured and unstructured spatial effects for the probability parameter, and based model choice on deviance information criterion (DIC). Model fit was further assessed through the plots of the observed and predicted values. The details of the implemented models including the values of the DIC are presented in Table 1. For all models, we performed 12 000 iterations with 2000 set as burn-in and the thinning parameter set at 10. The generated Markov chains were investigated through trace plots to ascertain mixing and convergence. Trace plots for some of the parameters and those of the observed against fitted values are presented in Appendix 2.

**Table 1.** Assessment of various model specification used in this study^a^

Model	Specification	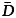	pD	DIC
1	Spatial (*S*_str_ + *S*_unstr_) + ST	12 013.66	219.49	12 452.66
2	Trend + ST	9661.47	317.98	10 297.43
3	Trend + spatial (*S*_str_ + *S*_unstr_)	12 237.92	107.30	12 452.53
4	Trend + spatial (*S*_str_ + *S*_unstr_) + ST	9134.13	215.38	9564.89

a *S*_str_ represents structured spatial random effect, *S*_unstr_ is the unstructured random effect, Trend is the temporal term and ST accounts for the spatio-temporal random effect.

## Results

### Preliminary COVID-19 distribution in Africa

**Fig. 1.**
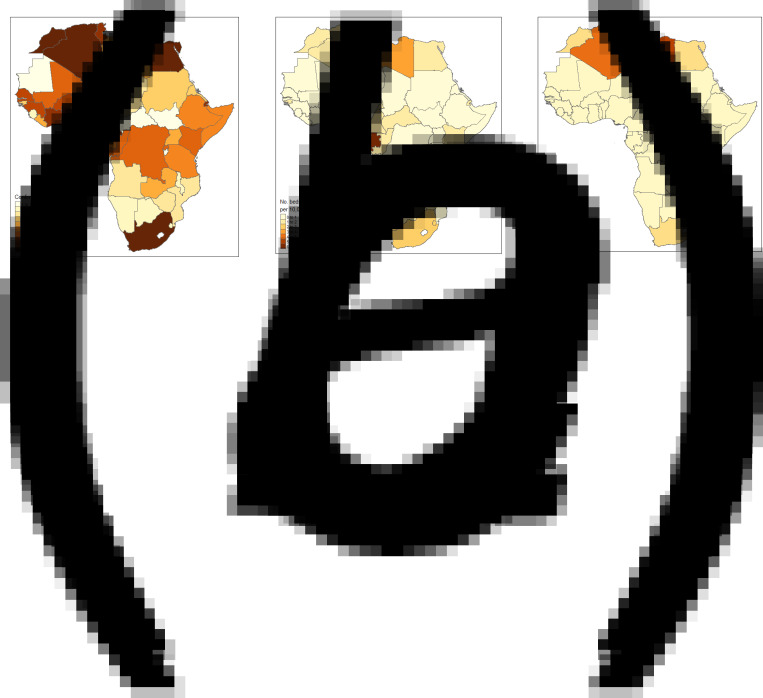
(a) Total number of confirmed COVID-19 cases as of 11 April 2020, (b) Distribution of the number of hospital beds (per 10 000), (C) Distribution of the number of physicians (per 10 000).

**Fig. 2.**
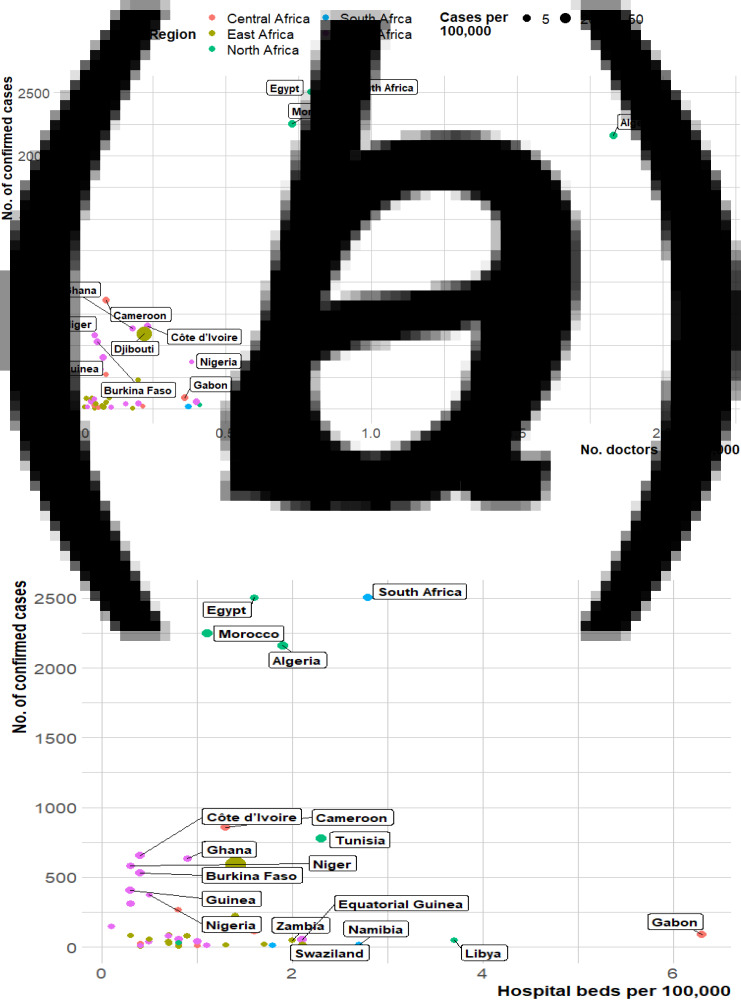
Scatter plot of number of confirmed cases of COVID-19 and healthcare capacities (Number of hospital beds/medical doctors).

**Fig. A3.**
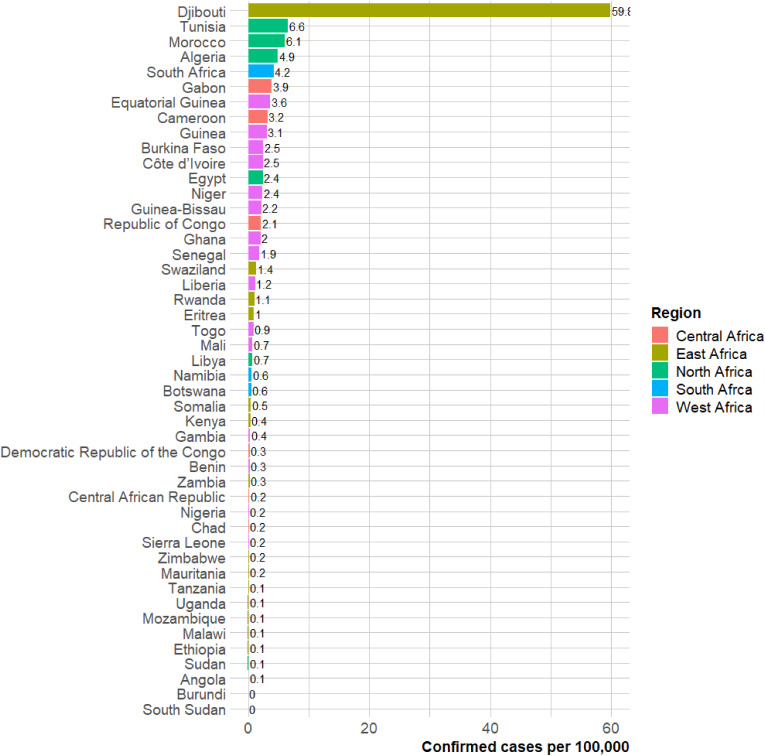
Burden (cases per 100,000 population) of COVID-19 across Africa as at 16th April 2020.

The distribution of COVID-19 cases as of 11 April and the number of hospital beds and physicians (per 10 000 population) by country are presented in Figure 1(a–c). The figure shows that cases of COVID-19 varied geographically across Africa with notable high incidence in West and North Africa (Fig. 1a). However, when this incidence was converted to cases per 100 000 population, the burden of the disease in Africa was greatest in Djibouti, East Africa and North Africa (Tunisia, Morocco and Algeria) (Figure A3, Appendix 3). Interestingly, countries with the highest burden of the pandemic in Africa are among those with the highest number of hospital beds and physicians, particularly those from the northern fringe (Fig. 1b, c). This could be due to the testing capacities of these countries as it is expected that countries with better healthcare capacity would be able to conduct more tests and thus able to detect more cases. Figure 2 examines the pattern of relationships between the pandemic and numbers of hospital beds and physicians. Findings reveal a positive correlation between COVID-19 and each of number of physicians (*r* = 0.49, *P*-value 0.001) and hospital beds (*r* = 0.14, *P*-value = 0.34) though only the estimate for physicians is statistically significant.

### Spatio-temporal analysis

**Fig. 3.**
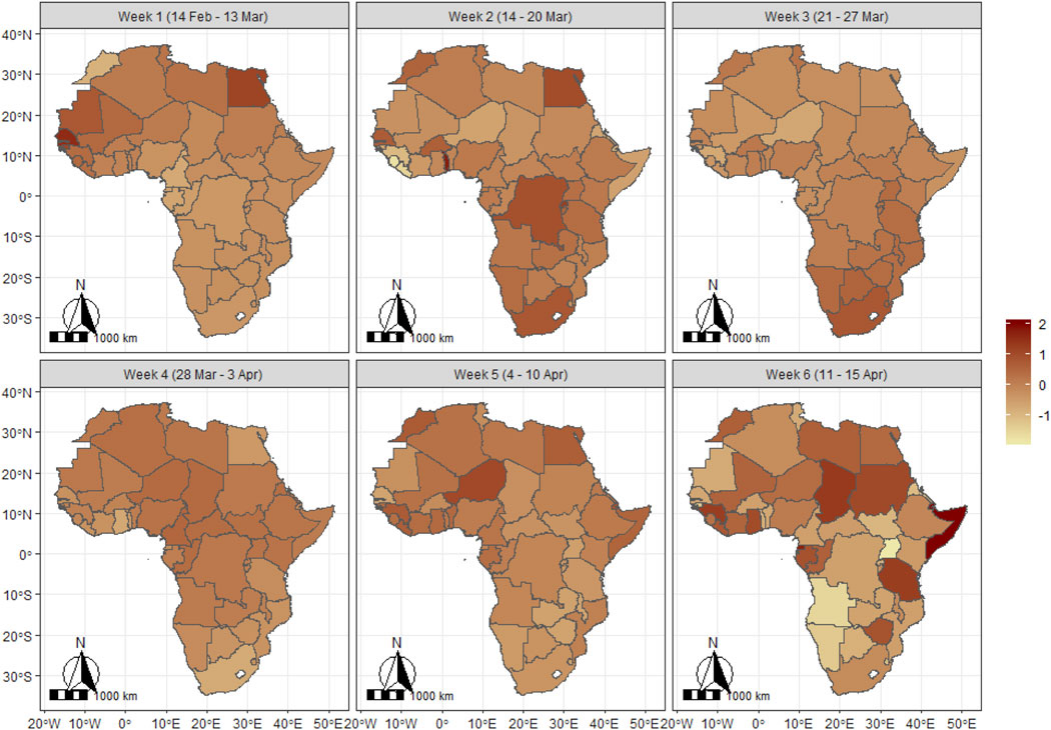
Spatiotemporal pattern of COVID-19 in Africa based on expected value of the Poisson parameter (mu (*μ*) parameter). The scales indicate the range of the posterior mean estimates of the parameter.

Table 1 presents the specifications of the four models considered together with the values of the model diagnostics criteria. As is evident from Table 1, the fourth model whose mean component includes the trend, structured and unstructured random effects and the spatio-temporal components had the lowest DIC value and thus provides the best fit. Presentations of results shall therefore be based on those of this model. Figure 3 presents the maps of Africa showing the spatio-temporal patterns of the parameter *μ*, measuring the frequency of occurrence of COVID-19 on the continent during the period 14 February to 15 April 2020, based on a 6-week group. The results show that during the period 14 February–13 March, Senegal had the highest average record of the pandemic closely followed by countries such as Egypt and Mauritania. By the week of 14–20 March, the burden shifted to Togo, South Africa, Egypt, DR Congo, Senegal and Burkina Faso. However, during the period 21–27 March, South Africa had the highest burden of the pandemic, while for the week 28 March to 3 April, the burden of the pandemic appears to be relatively similar across most of the African countries. For the week 4–10 April, the burden shifted to Niger, Morocco, Guinea, Egypt and Sierra Leone and lastly, for the week 11–15 April, the burden was most felt by countries such as Somalia, Chad, Guinea, Tanzania, Gabon, Sudan and Zimbabwe but least for Namibia, Angola and Uganda.

**Fig. 4.**
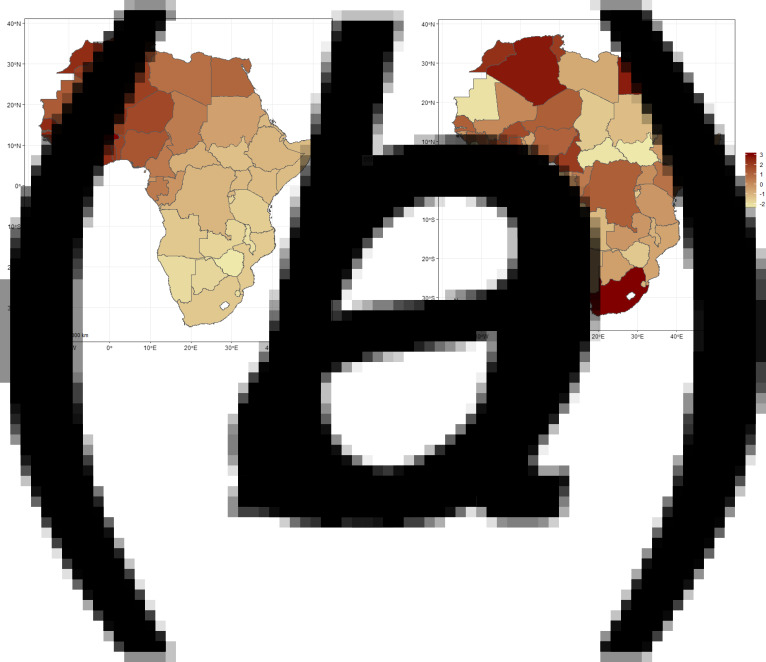
Structured (a) and unstructured (b) spatial effects for the mean of COVID-19 (mu (*μ*) parameter) in Africa. The scales indicate the range of the posterior mean estimates of the parameter. Note: The structured spatial map was obtained from a component of the model that assumes spatial correlation among the countries implying that neighbouring countries can influence events among themselves which is not the case for two countries that are at distance and share no boundary. The unstructured map assumes independence among the countries.

Results for the structured and unstructured random terms are presented in Figure 4a, b for the frequency of occurrence of the pandemic. The two maps reveal different patterns across the continent, which could have been the result of the neighbourhood structures of the countries that were taken into account in the structured effect. Thus, the structured random effect presents a western-southern divide indicating that the burden of the pandemic has been generally heavier among countries in the West African region specifically, in neighbouring Ivory Coast, Burkina Faso, Ghana, Mali, Guinea, Senegal, as well as Morocco and Algeria in North Africa but generally lesser in the southern African countries. However, estimates from the unstructured effect that assumes independent and identically distributed normal prior show that South Africa, Egypt, Algeria, Morocco, Tunisia and Cameroon had the highest individual burden but it was lowest for South Sudan, Central African Republic and Mauritania. The estimates are moderate for Nigeria, Ghana, Ivory Coast, Burkina Faso, Niger, Senegal, Republic of Congo and Kenya.

**Fig. 5.**
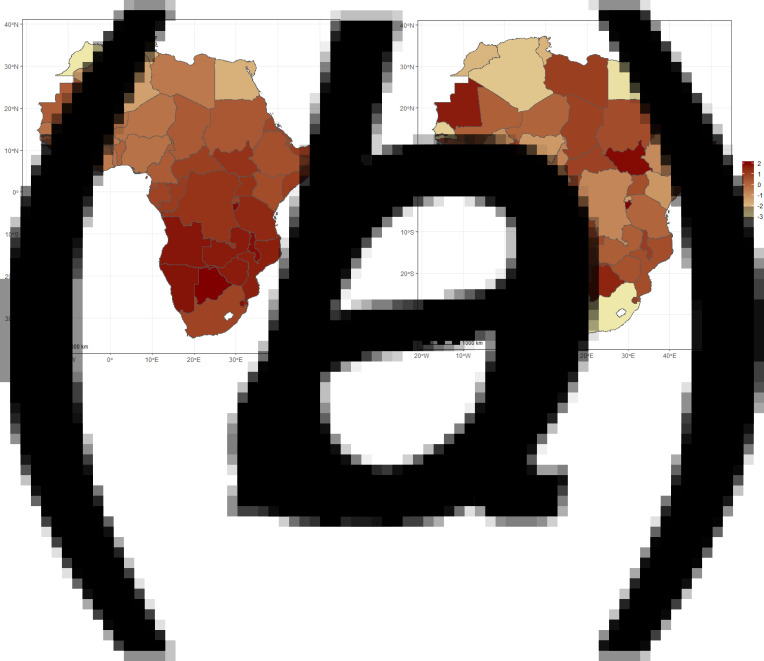
Structured (a) and unstructured (b) spatial effects for the probability of no occurrence of COVID-19 (*π* parameter) in Africa. The scales indicate the range of the posterior mean estimates of the parameter.

**Fig. 6.**
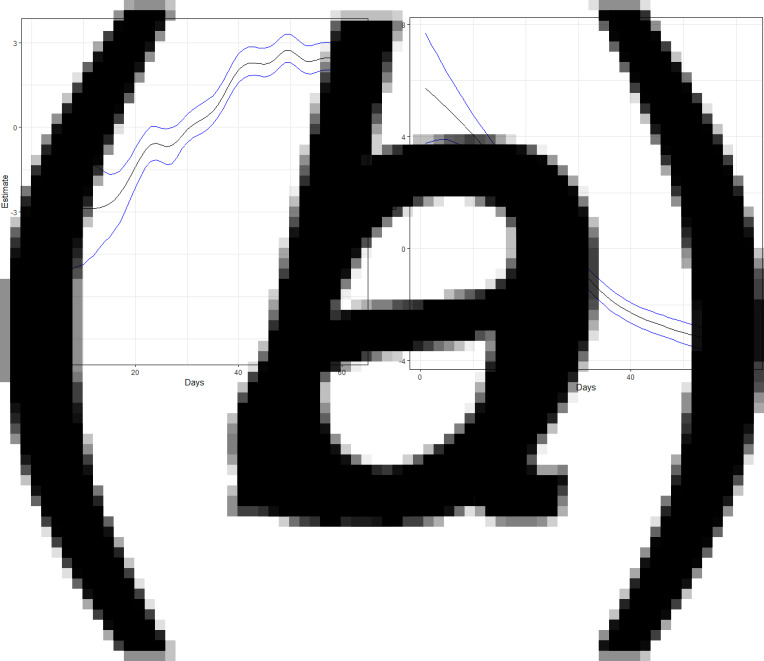
Temporal trend of COVID-19 for (a) mean number of occurrence and (b) likelihood of no occurrence.

Results for the spatial patterns of the probability of no occurrence are presented in Figure 5a, b. The structured spatial effects show that neighbouring countries in southern and central Africa had the highest likelihood of not recording any cases of COVID-19. The map for the unstructured effect however reveals that the likelihood of not reporting a case was highest among Mauritania, Botswana, South Sudan, Burundi, Namibia, Libya, Chad, Central African Republic, Somalia, Malawi, Benin, Sierra Leone, The Gambia and Swaziland, but lowest for South Africa, Egypt, Algeria, Morocco, Tunisia and Senegal. The temporal patterns presented in Figure 6a, b displayed the posterior mean estimate (black) and 95% credible interval (blue). The figure reveals that the frequency of occurrence has been on a consistent rise since the first case was reported up to around day 50, followed by a somewhat gradual decline for about 3 days after which there was evidence of another rise. On the other hand, the estimates for the likelihood of no occurrence decline sharply until day 50 and appear to flatten thereafter. Note that the wide credible intervals for the early days are evidence of few reported cases during this period.

## Discussion

This study has established that the burden of COVID-19 in Africa varies geographically with each country's healthcare-related variables. As COVID-19 causes significant health and economic challenges globally, the impacts on Africa are still in their infancy. The present study reveals the geographical spread of the disease in Africa and its relation to individual country health capacities. Findings from the spatio-temporal analysis reveal that the occurrence and burden of COVID-19 in Africa varied geographically in neighbouring countries particularly in the western part of the continent which could imply that neighbouring countries pose significant importation risk to each other. This is quite a challenging problem due to the free movement of people within the region and supports the contention that African countries should form a coalition to fight against COVID-19.

There are several possible reasons for the geographical distribution of COVID-19 across Africa. The first is the route of disease introduction to Africa. For example, when China was the only COVID-19 epicentre, the risk of COVID-19 importation from China to Africa was high for North Africa [5]. Previous studies based on travel data from provinces in China identified Egypt, Algeria and South Africa as having the highest importation risk of first cases from China [4, 5]. However, as the epicentre changed from China to Italy and the USA, the risk to other regions in Africa increased as there were more African travellers from Europe and North America than Asia [4]. The second reason is the issue of border porosity in most African countries [22, 23]. The ease of people's movement over borders could increase importations as seen in Nigeria where many Nigerians returning from the Ivory Coast were diagnosed to have COVID-19. This is what happens with neighbouring African countries as they endanger each other unless enhanced border and flight restrictions are put in place.

COVID-19 poses significant health issues because it can quickly overwhelm healthcare capacity if not checked. In this study, countries with greater healthcare capacity measured by number of hospital beds and physicians had more cases. This seeming paradox could be explained as healthcare capacity has been used as a measure of a country's wealth [24, 25]. Additionally, previous studies using air travel volume data have suggested some African countries to be at higher risk of COVID-19 importation from Europe and China [4, 5]. Therefore, it is more likely that citizens of such countries will travel overseas. This tendency increases the chance of importing COVID-19 on their return. Our findings are consistent with a previous study that suggested African countries with more sophisticated surveillance systems are more likely to identify a higher risk of disease importation [5]. This implies that additional public health capacity is needed for those countries with limited resources to detect COVID-19 and undertake meaningful contact tracing to curtail the rapid spread of the virus.

There have been warnings that some countries in Africa could be the next COVID-19 epicentre [7, 26]. Thus far, the burden of COVID-19 in Africa is low in comparison with Europe, Asia and the Americas. There is a pressing need for early introduction of interventions such as isolation, quarantine and social distancing [27]. However, many African countries are poor and whether these control measures will work as effectively as seen in China is still an open question [26].

It is worth nothing that the findings reported in this study are based on data available on 15 April 2020 and, at this time, the pandemic was in its early stages in Africa. Consequently, future spatial patterns could change considerably as more testing is done and new cases detected. Also, considering the low testing capacities of most African countries, it is very likely that the reported data may not truly reflect the extent of the pandemic on the continent due to underascertainment of cases. Additionally, the data on healthcare capacity may not be exactly comparable across countries due to data sources and means of monitoring. Notwithstanding these comments, the spatio-temporal maps generated in this study provide early evidence of the distribution of the situation on the African continent over the period considered.

## Conclusion

As the pandemic spreads, the African Centers for Disease Control have intensified investment in enhancing diagnostic and surveillance capacity across the various African countries [7]. Africa may only be able to counter this virus if conscientious efforts and support are garnered globally to battle COVID-19 [7]. Unsurprisingly, we have shown the trajectory of COVID-19 in Africa is increasing, with each African country posing a risk to its neighbours. The findings in this study will be useful in implementing targeted intervention strategies based on heterogeneity of the disease patterns and optimal allocation of limited resources.

## Figures and Tables

**Fig. A1.**
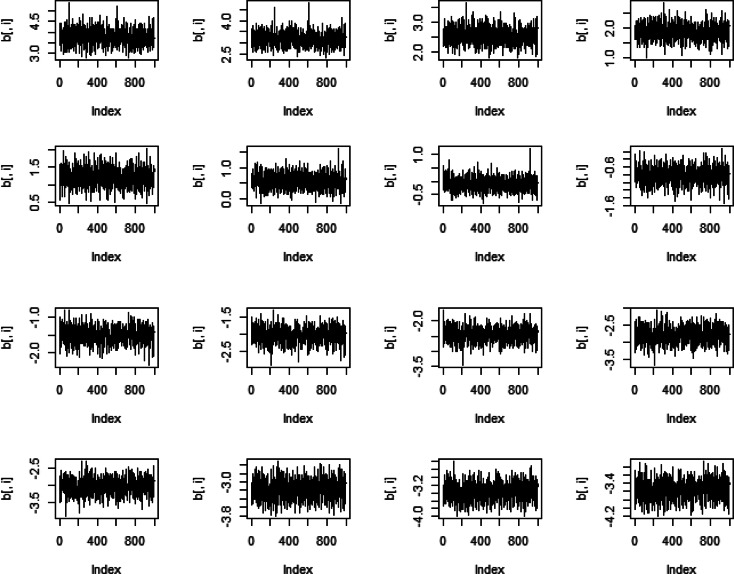
Trace plot for some of the parameters.

**Fig. A2.**
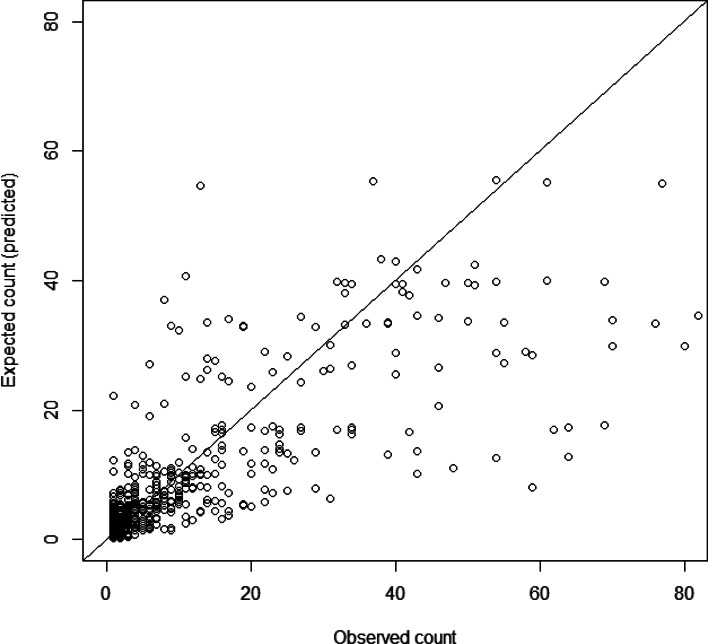
Plot of observed and expected (predicted) non-zero cases.

## Data Availability

The specific data and codes used for this project are available at https://github.com/OsafuAugustine/myfirstrepo/tree/master/Ezra.

## Appendix 1

Parameter estimation of the hurdle Poisson model was through a hierarchical Bayesian approach where prior distributions are specified to the different parameters and functions of the model. For the structured spatial random component, a correlation structure is assumed for the spatial structured based on their spatial proximity and a Markov random field [20] was considered as a prior distribution for the discrete sets of spatial locations such that separate regression coefficients correspond to the distinct countries. The neighbourhood structure of the countries determines the Markovian structure where proximate locations that share boundary are assigned a weight of 1 and 0 if they do not. The precise form of the prior distribution is specified by the following Gaussian structure

where *N*_*s*_ = |*δ*_*s*_| is the number of adjacent neighbours, *rεδ*_*s*_ indicates that country *r* is a neighbour to country *s* and is the variance component that ensures the spatial smoothness. The conditional mean of *β*_str,*s*_ given all the other coefficients is the average over the neighbouring countries.

For the unstructured spatial effects, the spatial proximity (neighbourhood structure) of the countries were ignored and the countries are considered as membership representing different groups, and an exchangeable independent and identically distributed Gaussian prior with the following property *f*_unstr_(*s*) ~ *N*(0, *τ*_unstr_) was considered. Both variances were assigned inverse gamma prior.

The temporal component was modelled through a Bayesian P-spline prior as proposed by [19]. The prior allows for non-parametric estimation of the temporal function as a linear combination of basis function (B-splines) expressed as

where *B*_*t*_(*z*) are B-splines, and the coefficients *α*_*t*_ are defined to follow a first- or second-order Gaussian random walk smoothness prior for which the second order used in this study is defined as

where the error term *ɛ* is assumed to be independently and identically distributed *ɛ* ~ *N*(0, *τ*^2^). Again, the variance, *τ*^2^ controls the smoothness of the function and it is jointly estimated with the basis function coefficients by assigning a weakly informative inverse gamma prior such that *τ*^2^ ~ *IG*(*ɛ*, *ɛ*). A cubic spline with 20 inner knots were considered as based on previous studies, these would yield sufficient flexibility to capture the non-linearity in the temporal pattern [19, 28].

As a result of the complex nature of distributional regression model, the posterior distribution leads to full conditionals for the unknown regression coefficients that are analytically intractable. Consequently, fully Bayesian inference was based on Markov chain Monte Carlo technique (MCMC) to generate samples from the full conditional and used for posterior estimation. The MCMC sampler was executed as a Metropolis-Hastings algorithm through iterative weighted least square (IWLS) as developed by [17]. For all the models considered, we generated 12 000 iterations and set the burn-in as 2000 and then thin every 10^th^ observation for parameter estimation.
